# Weiterbildung Urologie in Deutschland, Österreich und der Schweiz (DACH-Raum) unter der Lupe: Unterschiede, Gemeinsamkeiten, Vor- und Nachteile und Zukunftsperspektiven

**DOI:** 10.1007/s00120-023-02058-9

**Published:** 2023-03-20

**Authors:** Philippe-Fabian Pohlmann

**Affiliations:** Medizinische Fakultät, Klinik für Urologie, Universitätsklinikum Freiburg, Hugstetterstraße 55, 79106 Freiburg, Deutschland

**Keywords:** Weiterbildung, Ausbildung, Prüfung, Zulassung, Facharzt, Residency, Training, Education, Licensing, Specialization

## Abstract

**Hintergrund:**

Die Facharzttitel werden im DACH-Raum (Deutschland, Österreich und Schweiz) gegenseitig anerkannt. Während der Weiterbildung bestehen jedoch deutliche Unterschiede in Struktur, Organisation und Inhalt.

**Ziel der Arbeit (Fragestellung):**

Die Organisation, Formalitäten und Zuständigkeiten sowie Zulassungsbedingungen für die abschließende Prüfungen, insbesondere der Prüfungsformate in den DACH-Ländern, sollen untersucht werden. Zudem sollen diese kritisch aus dem Blickwinkel moderner medizinischer Ausbildung beleuchtet werden.

**Material und Methoden:**

Es wurden öffentlich im Internet zugänglichen Dokumente der zuständigen Autoritäten für die Ärztliche Weiterbildung im DACH-Raum analysiert. Außerdem erfolgte eine narrative Durchsicht der Literatur in medizinischen Datenbanken zum Thema Ärztliche Weiterbildung in der Urologie.

**Ergebnisse:**

Die Mindestweiterbildungsdauer beträgt 5 (D) bis 6 Jahre (A, CH). Eine (chirurgische) Basisausbildung ist in Österreich und der Schweiz obligat. In der Schweiz muss die Ausbildungsstätte mindestens einmalig gewechselt werden. Eine Weiterbildung in Teilzeit ist in allen Staaten möglich. Forschung und Teilnahme an Jahreskongressen sind nur in der Schweiz verpflichtend. Nur in der Schweiz sind formative Prüfungen vorgesehen. In allen Ländern ist mindestens eine summative Prüfung zur Erlangung des Facharzttitels notwendig. In Österreich und der Schweiz muss der schriftliche Teil der EBU-Prüfung (European Board of Urology) bestanden werden.

**Diskussion:**

Die Schweiz bietet und fordert aus medizindidaktischer Sicht aktuell die meisten modernen Elemente in der Weiterbildung. Die zertifizierte strukturierte Weiterbildung nach Vorbild der Deutschen Gesellschaft für Urologie (DGU; Weiterbildungscurriculum Urologie, WECU) integriert moderne Ansätze in Deutschland. Mit der zeitnahen Anwendung von z. B. EPA („entrustable professional activities“) könnte die deutschsprachige Urologie international zu den Vorreiternationen und -fachdisziplinen gehören.

## Abstract (en)

**Background:**

Specialist titles are mutually recognized in the DACH (Germany [D], Austria [A], and Switzerland [CH]) region. During continuing education, however, there are significant differences in structure, organization and content.

**Objectives:**

Organization, formalities and responsibilities, as well as admission requirements for the final examinations, in particular the examination formats in the DACH countries will be examined. In addition, these will be critically examined from the perspective of modern medical education.

**Materials and methods:**

Documents publicly available on the Internet from the responsible authorities for continuing medical education in the DACH region were analyzed. In addition, a narrative review of the literature in medical databases on the topic of continuing medical education in urology was performed.

**Results:**

The minimum duration of residency is 5 (D) to 6 years (A, CH). Basic (surgical) training is mandatory in Austria and Switzerland. In Switzerland, the training site must be changed at least once. Part-time training is possible in all countries. Research and participation in annual congresses are mandatory only in Switzerland. Formative examinations are only required in Switzerland. In all countries, at least one summative examination is required to obtain the specialist title. In Austria and Switzerland, the written part of the European Board of Urology (EBU) examination must be passed.

**Conclusion:**

From a medical education perspective, Switzerland currently offers and demands the most modern elements in specialist training. The certified structured continuing education modelled on the “Deutschen Gesellschaft für Urologie” (DGU; “Weiterbildungscurriculum Urologie”, WECU) in Germany integrates modern approaches in Germany. With timely implementation of, for example, entrustable professional activities (EPAs), urology in German-speaking countries could be pioneering nations and disciplines internationally.

## Kurze Hinführung zum Thema

Die Weiterbildung in der Urologie unterliegt auch in Deutschland, Österreich und der Schweiz, hier vereinfacht DACH-Raum (D = Deutschland, A = Österreich, CH = Schweiz) genannt, einem stetigen Wandel. In Deutschland haben mit der Einführung der Weiterbildungsordnung von 2020 Kompetenzniveaus Einzug gehalten und bloße Operationszahlen verloren an Gewicht. In der Schweiz sind neue Konzepte wie „entrustable professional activities“ (EPA) in vielen Bereichen der medizinischen Ausbildung bereits im Alltag angekommen. Und die Kolleg:innen in Österreich lernen bereits seit einigen Jahren auf die europäische „European Board of Urology“ (EBU)-Prüfung, um selbstständig praktizieren zu dürfen. Diese Unterschiede und Gemeinsamkeiten sollen in dieser Arbeit untersucht, eingeordnet und aus der Sicht moderner Medizindidaktik analysiert werden.

## Hintergrund

Trotz der weltweiten Coronapandemie in den vergangenen Jahren liegt die Mobilität aufgrund der Personenfreizügigkeit innerhalb der Europäischen Union (EU) insgesamt auf einem stabilen und hohen Niveau [19]. Zwischen den Mitgliedern der EU Deutschland und Österreich herrschen freie Mobilität und Erwerbstätigkeit. Mit dem Nicht-EU-Mitglied Schweiz bestehen Verträge mit der EU, welche die Personenfreizügigkeit nahezu uneingeschränkt erlauben [14].

Im DACH-Raum erleichtern zusätzlich die räumliche Nähe, die (meist) gleiche Sprache und die historisch gewachsene Zusammenarbeit der zuständigen medizinischen Fachgesellschaften die Fluktuation des medizinischen Personals. Dies belegen auch erhobene Zahlen: Bei der Abwanderung deutscher Ärzt:innen ins Ausland liegen die Schweiz und Österreich mit deutlichem Abstand auf den ersten beiden Plätzen. Umgekehrt ist für Kolleg:innen aus Österreich Deutschland die erste Adresse für einen Landeswechsel. Im Jahr 2021 wanderten 167 Ärzt:innen aus Österreich nach Deutschland ein. Davon waren 161 ohne Gebietsbezeichnung, was annehmen lässt, dass es sich größtenteils um Kolleg:innen direkt nach dem Studium (davon viele deutsche „Numerusclaususflüchtlinge“) oder in der Weiterbildung befindliche Ärzt:innen handelt [20].

**Figure 1:**
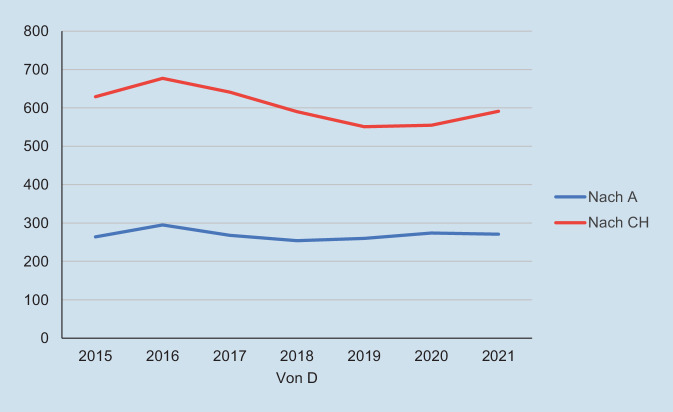

Im DACH-Raum sind Facharzttitel und Arztdiplome gegenseitig anerkannt [6]. Aber bereits vorher, innerhalb der Facharztausbildung, wird der Wechsel über die Landesgrenzen hinweg immer häufiger. Genaue Zahlen zu den Wechseln innerhalb der Weiterbildung oder den Gebietsbezeichnungen der aus- und eingewanderten Ärzt:innen geben die allgemeinen Statistiken (z. B. der Bundesärztekammer) zumindest bislang nicht wieder. Es ist aber davon auszugehen, dass diese mindestens parallel zur allgemeinen Mobilität über die Landesgrenzen hinweg verlaufen (Abb. 1).

Die Facharztweiterbildung für Urologie im DACH-Raum ist in ihren Grundzügen sehr ähnlich, unterscheidet sich aber dennoch in einigen bedeutenden Merkmalen.

Für Weiterzubildende ist es oft nicht einfach, die notwendigen Informationen für einen Landeswechsel zu finden. Auch für Weiterbilder:innen und andere Fachkolleg:innen bleibt oft unklar, welche Anforderungen ein Wechsel während der Weiterbildung mit sich bringt.

Zudem wirft die gegenseitige Anerkennung der Qualifikationen unweigerlich auch Fragen auf: Können sich Patient:innen und Arbeitgeber:innen wirklich darauf verlassen, dass im Ausland weitergebildete Ärzt:innen den lokal geforderten Qualifikationen gerecht werden können? Wie vergleichbar sind die Weiterbildungen wirklich?

## Ziele der Arbeit

Die vorliegende Arbeit soll einen strukturierten und möglichst vollständigen Überblick über die Organisation, Formalitäten und Zuständigkeiten während der Weiterbildung im Fach Urologie in den DACH-Ländern bieten. Zudem sollen markante Unterschiede in den Inhalten der verschiedenen Weiterbildungsordnungen (WBO) aufgezeigt werden. Schließlich werden die Zulassungsbedingungen für die abschließende Prüfung untersucht und verglichen. Dabei liegt ein besonderes Augenmerk auf den genutzten Prüfungsformaten. Die Ergebnisse sollen dann aus dem Blickwinkel moderner medizinischer Ausbildung und im internationalen und fächerübergreifenden Vergleich bewertet werden.

## Material und Methoden

### Ethische Überlegungen

Ein Votum einer Ethikkommission wurde nicht eingeholt. In der vorliegenden Arbeit wurden ausschließlich öffentlich zugängliche Informationen verwendet. Die Studie entspricht der Deklaration der WHO von Helsinki [4].

### Literaturrecherche

Der Autor analysierte die öffentlich im Internet zugänglichen Dokumente der zuständigen Autoritäten für die Ärztliche Weiterbildung in Deutschland, Österreich und der Schweiz (Stand 09/2022). Außerdem wurden öffentliche Daten wie z. B. die Ärztestatistik der Bundesärztekammer Deutschland verwendet. Anschließend erfolgte eine narrative Durchsicht der Literatur in den Datenbanken MEDLINE, Google Scholar und Web of Science bis 09/2022 zum Thema Ärztliche Weiterbildung generell und speziell in der Urologie. Die Ergebnisse wurden qualitativ zusammengefasst [12].

## Ergebnisse

### Zuständigkeiten in der Weiterbildung

In Deutschland sind die Landesärztekammern für die ärztliche Weiterbildung zuständig. Die jeweiligen (Muster‑)Weiterbildungsordnungen werden von der Bundesärztekammer (BÄK) beschlossen und dann von den Landesärztekammern umgesetzt [21].

Ärzt:innen in Österreich sind in der Österreichischen Ärztekammer (ÖÄK) organisiert und diese ist auch über die Österreichische Akademie der Ärzte (ÖAÄ) zuständig für die ärztliche Weiterbildung [22].

In der Schweiz ist das Schweizerische Institut für ärztliche Weiter- und Fortbildung (SIWF) zuständig für die Organisation der ärztlichen Weiterbildung. Sie ist ein autonomes Organ des ärztlichen Berufsverbands *Foederatio Medicorum Helveticorum* (FMH; [23]; Tab. 1).

**Table 1:** 

	D	A	CH
Organisation/Zuständigkeit	Föderal (LÄK)	National (ÖAÄ/ÖÄK)	National (SIWF/FMH)
Weiterbildungsdauer (min.)	5 Jahre	6 Jahre	6 Jahre
(Chirurgische) Basisweiterbildung	Fakultativ, maximal 1 Jahr	Obligat, 9 Monate	Obligat, 1 Jahr
Nicht-fachspezifische Anerkennung	Fakultativ, maximal 1 Jahr	Nein	Fakultativ, maximal 1 Jahr
Weiterbildungsstätten Urologie (min.)	1	1	Minimal 2 (minimal 2 Jahre A1/A2, davon minimal 1 Jahr A1)
Teilzeit	Möglich	Möglich, minimal 50 % in Lehrambulatorien	Möglich, minimal 50 %
Anerkennung ausländischer Weiterbildungen	Möglich, auf Antrag	Möglich, auf Antrag	Möglich, minimal 2 Jahre in CH
Forschungsanerkennung	Maximal 6 Monate	9 Monate (wissenschaftliches Modul)	Maximal 1 Jahr
Publikation	Fakultativ	Fakultativ	Obligat (1)
Jahresversammlungen	Fakultativ	Fakultativ	Minimal 3 Teilnahmen mit 2 Präsentationen

*D* Deutschland, *A* Österreich, *CH* Schweiz

### Organisation der Weiterbildung

#### Weiterbildungsdauer

In allen Ländern des DACH-Raums geben die zuständigen Organe eine Mindestdauer für die Weiterbildung vor. Während in Deutschland die Mindestdauer 5 Jahre beträgt, dauert die Weiterbildung in Österreich und der Schweiz jeweils mindestens 6 Jahre.

Die Wahl der Weiterbildungsstätte(n) ist in allen drei Staaten frei und obliegt den Weiterzubildenden. Jedoch müssen die Weiterbildungsstätten jeweils von den Dachverbänden (LÄK, ÖÄK, SIWF) anerkannt sein.

#### Weiterbildungsstruktur

In Deutschland ist auf dem Weg zum Facharzttitel keine Ausbildung in Fächern außerhalb der Urologie (mehr) vorgesehen. In Österreich ist die Basisausbildung mit einer Dauer von 9 Monaten für alle Fachgebiete obligat. Hierfür bietet sich für die Weiterbildung in Urologie eine chirurgische Basisausbildung an. In der Schweiz muss zunächst ebenfalls eine chirurgische Basisausbildung mit einer Dauer von einem Jahr erfolgen. Die Ausbildung ist in Österreich und der Schweiz möglich in Abteilungen der Chirurgie, Allgemeinchirurgie, Traumatologie, Viszeralchirurgie oder Gefäßchirurgie.

Die Weiterbildung Urologie ist in Österreich aufgegliedert in eine 3‑jährige Grundausbildung und 27-monatige Schwerpunktausbildung. Zur Auswahl stehen in der Schwerpunktausbildung 7 Module, wobei 3 Module auszuwählen sind.

In der Schweiz und in Deutschland kann optional ein Jahr nicht-fachspezifische Ausbildung anerkannt werden. Die möglichen Fächer sind in beiden Ländern jeweils vorgegeben. In Österreich ist die Anerkennung einer nicht-fachspezifischen Ausbildung, abseits der chirurgischen Basisausbildung, nicht vorgesehen.

**Figure 2:**
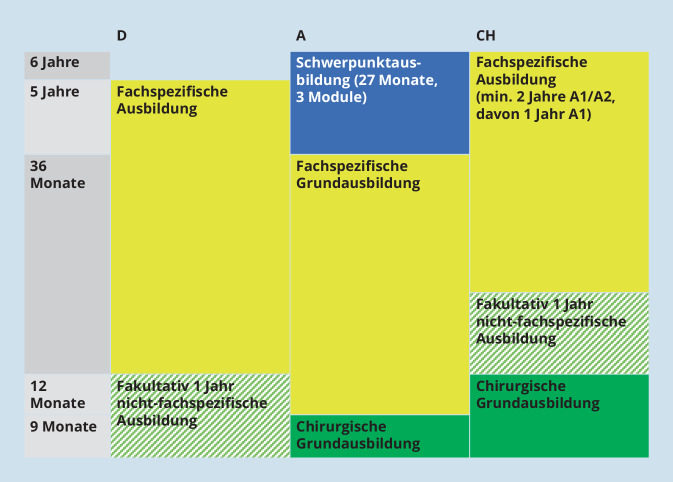

Während in Deutschland und Österreich die fachspezifische Weiterbildung im Fach Urologie an einem einzigen (geeigneten) Standort erfolgen kann, gibt es in der Schweiz genauere Vorgaben. Hier müssen mindestens 2 Jahre an einer Weiterbildungsstätte der Kategorie A1 oder A2 absolviert werden, davon mindestens ein Jahr Kategorie A1. Stark vereinfacht entspricht Kategorie A1 dem Vollangebot des urologischen Spektrums, Spitäler mit geringerem Behandlungsumfang werden nach WBO der Schweiz abgestuft in A2 oder B1 kategorisiert [23]. Gleichzeitig darf die Weiterbildung nur maximal 4 Jahre an einem Haus der Kategorie A1 oder A2 erfolgen und maximal ein Jahr in Kategorie B1. Weiterhin muss mindestens ein Jahr der Weiterbildung in Urologie an einer zweiten Weiterbildungsstätte absolviert werden (Abb. 2).

#### Teilzeit und Anerkennung von Weiterbildung im Ausland

Eine Weiterbildung in Teilzeit ist in allen Ländern möglich. In der Schweiz ist ein Mindestarbeitspensum von 50 % (= Wochenarbeitszeit 22,5 h) vorgesehen. In Österreich muss abhängig von der Ausbildungsstätte bei einer Wochenarbeitszeit von 35 h ein Mindestpensum absolviert werden: Krankenanstalten 12 h/Woche, Lehrpraxen/Lehrgruppenpraxen 15 h/Woche, Lehrambulatorien 17,5 h/Woche. In Deutschland bestehen keine näheren Vorgaben diesbezüglich.

Bei einem Wechsel aus dem Ausland in die Schweiz während der Weiterbildungszeit müssen noch mindestens 2 Jahre an einer anerkannten Schweizer Weiterbildungsstätte erfolgen. In Österreich und Deutschland kann eine Anerkennung von im Ausland erfolgter Weiterbildung erfolgen, diese ist jedoch im Einzelfall zu prüfen. Eine Mindestausbildungszeit im Zielland ist nicht vorgeschrieben.

#### Forschung, Publikationen und Jahresversammlungen

Aktive Forschung wird in allen Ländern des DACH-Raums als Weiterbildungszeit anerkannt. Die Dauer unterscheidet sich hier. Während die deutschen Landesärztekammern in der Regel maximal 6 Monate Forschungszeit anerkennen, darf in der Schweiz bis zu einem Jahr geforscht werden. In Österreich kann im Rahmen der Schwerpunktausbildung ein wissenschaftliches Modul gewählt werden. Dieses Modul ermöglicht dann eine Anerkennung von 9 Monaten Forschungszeit.

Die Weiterbildungsordnung der Schweiz macht bezüglich der Teilnahme an nationalen Jahresversammlungen und Publikationen die genausten Vorgaben: Während der Weiterbildung muss mindestens 3‑mal an der nationalen Jahresversammlung der Schweizerischen Gesellschaft für Urologie (SWISS UROLOGY) teilgenommen werden und es müssen mindestens 2 (Poster‑)Präsentationen erfolgen. Zudem muss in der Schweiz mindestens eine Publikation (mit *Peer Review*) während der Weiterbildung veröffentlicht werden. Die WBO in Deutschland und Österreich stellen Teilnahmen an Kongressen, Publikationen und Präsentationen frei.

### Inhalte der Weiterbildungen

Die während der Weiterbildung zu erlangenden Fertigkeiten und Kompetenzen sind in Deutschland von den Landesärztekammern, in Österreich von der ÖÄK und in der Schweiz vom SIWF festgelegt. Diese Kataloge sind in allen Ländern recht allgemein gehalten und sollen als Grundlage für individuelle Weiterbildungskonzepte der lokalen Weiterbildungsstätten dienen.

Während in Österreich und der Schweiz Wissen und Fertigkeiten relativ unspezifisch aufgelistet werden, hat sich dies mit der neuesten Weiterbildungsordnung von 2020 in Deutschland verändert: Statt einer einfachen Liste mit Themen und Zahlenwerten zu Untersuchungen und Fertigkeiten werden Kompetenzniveaus definiert. Zusätzlich zu den Richtzahlen für Untersuchungen und Prozeduren wird zwischen kognitiven Kompetenzen und Methodenkompetenzen (Niveaus: 1. benennen und beschreiben, 2. systematisch einordnen und beschreiben) und Handlungskompetenzen (Niveaus: 1. unter Anleitung durchführen, 2. selbstverantwortlich durchführen) unterschieden. Das Kompetenzniveau muss dann durch die Weiterbilder:in bestätigt werden.

Allen Katalogen ist gemein, dass sie zunächst auf die allgemeine fachurologische Ausbildung abzielen und das vollständige Spektrum der Urologie abdecken. Eine Besonderheit stellt die Weiterbildung in Österreich dar: Während in Deutschland und der Schweiz die Vertiefung spezieller Fertigkeiten und Zusatzbezeichnungen erst nach Erlangen des Facharzttitels vorgesehen ist, kann in Österreich im Rahmen der Schwerpunktausbildung bereits während der Weiterbildung eine punktuelle Spezialisierung erfolgen (Abb. 2). Neben dem wissenschaftlichen Modul können in der Schwerpunktausbildung noch Kinderurologie, Blasenfunktionsstörungen/Urodynamik, Andrologie, uroonkologische Chirurgie, minimalinvasive Techniken und Urogeriatrie gewählt werden [22].

Einen weiteren relevanten Unterschied gibt es in der Führung von Logbüchern. In Deutschland ist im Rahmen der Einführung der WBO 2020 und seit dem Beginn des Jahres 2021 das Führen eines elektronischen Logbuchs (eLogbuch) verpflichtend. Die Landesärztekammern stellen diese in ihren Online-Portalen zur Verfügung und Weiterzubildende und Weiterbildende haben jeweils Zugriff auf das eLogbuch. In Österreich und der Schweiz ist eine elektronische Dokumentation (noch) nicht vorgeschrieben, hier genügt die Dokumentation der geforderten Untersuchungs- und Operationszahlen auf Papier.

### Zulassungsvoraussetzungen und Prüfungen

Die Weiterbildungen in Urologie im DACH-Raum beinhalten je nach Land unterschiedliche Formen verpflichtender formativer und summativer Prüfungen [18]. Zudem müssen jeweils die notwendigen Zeiten (s. oben) erfüllt sein und Logbücher mit gewissen Vorgaben zur Durchführung von operativen Eingriffen und weiteren Kompetenzen vorgewiesen werden. Noten werden in keinem Land vergeben. Nur die Schweiz schreibt zudem die Durchführung formativer Prüfungen vor (Tab. 2).

**Table 2:** 

	D	A	CH
*(OP)-Logbuch*	Obligat	–	Obligat
*Arbeitsplatzbasierte Assessments*	–	–	4 pro Jahr
*Theoretisch-schriftliche Prüfungen*	–	Prüfung des European Board of Urology (EBU)^a^	Chirurgisches Basisexamen
			Prüfung des European Board of Urology (EBU)
*Praktisch-mündliche Prüfungen*	–	–	–
Dauer	Minimal 30 min		2 h
Operationssaal	–	–	1,5 h
Fallbesprechungen	30 min	–	30 min

^a^Gesonderte Auswertung (nur konforme Fragen)

*D* Deutschland, *A* Österreich, *CH* Schweiz

#### Logbücher

In allen Ländern des DACH-Raums müssen Logbücher geführt, Kompetenzen bescheinigt bzw. Richtzahlen für durchgeführte Untersuchungen und Eingriffe erfüllt, dokumentiert und nachgewiesen werden [23–25]. In der Regel müssen diese von den zuständigen Ausbilder:innen unterzeichnet werden und sind Grundlage für die Zulassung zur (abschließenden) summativen Prüfung.

#### Arbeitsplatzbasierte Prüfungen (formative Prüfungen)

Verpflichtende arbeitsplatzbasierte Prüfungen sind nur in der Schweizer Weiterbildung vorgesehen [26]. Das SWIF legt fest, dass mindestens vier praktische Prüfungen am Arbeitsplatz pro Jahr durchgeführt und dokumentiert werden müssen. Als Feedback-Instrumente sollen sie den Weiterbildungsstand erfassen und Grundlage für Mitarbeitergespräche bieten. Anerkannt von der SWIF sind die *Mini Clinical Evaluation Exercise* (Mini-CEX), die *Direct Observation of Procedural Skills* (DOPS) und die EPA [26]. Deutschland und Österreich machen hier (noch) keine Vorgaben.

#### Theoretisch-schriftliche Prüfungen (summative Prüfungen)

In Deutschland ist zur Erlangung des Facharzttitels Urologie keine schriftliche Prüfung notwendig.

Für Kolleg:innen in Österreich ist die erfolgreiche Teilnahme am ersten schriftlichen Teil der Prüfung des EBU obligat [27]. Bei der Auswertung der insgesamt 100 englischsprachigen „multiple choice questions“ (MCQ) wird jedoch auf Konformität mit der Österreichischen Approbationsordnung für Urologie geprüft und nur konforme Fragen werden gewertet. Die Prüfung ist computerbasiert und wird an geeigneten Prüfungszentren dezentral durchgeführt.

In der Schweiz sind insgesamt zwei summative Prüfungen auf dem Weg zum Facharzttitel zu bestehen. Zunächst muss das chirurgische Basisexamen bestanden werden. Diese MCQ-Prüfung wird auf Englisch durchgeführt und muss an einem festgelegten Ort in der Schweiz abgelegt werden [28]. Die zweite theoretisch-schriftliche Prüfung ist, wie in Österreich, die Prüfung des EBU. Im Gegensatz zu Österreich erfolgt jedoch keine gesonderte Auswertung der Fragen [29].

#### Praktisch-mündliche Prüfungen (summative Prüfungen)

In Österreich ist keine praktisch-mündliche Prüfung abzulegen.

In Deutschland stellt die praktisch-mündliche Prüfung den letzten Schritt zum Facharzttitel dar. Nach Erfüllung aller Zulassungsbedingungen (Logbuch, Zeugnisse etc.) erfolgt eine mindestens 30-minütige Prüfung, meist in den Räumlichkeiten der zuständigen Ärztekammer. Die Prüfung ist fallbasiert und wird von zwei Prüfer:innen aus dem Fachbereich abgenommen. Genaue Vorgaben zum Inhalt der Prüfung bestehen nicht.

Am umfangreichsten ist die praktisch-mündliche Prüfung in der Schweiz. Nach Bestehen der beiden schriftlichen Prüfungen (Basisexamen und EBU-Prüfung) kann die praktisch-mündliche Prüfung absolviert werden. Die Prüfung dauert ungefähr 2 h und ist in zwei Blöcke unterteilt. Zu einer 30-minütigen Fallbesprechung, ähnlich der Prüfung in Deutschland, kommen noch 90 min Beobachtung im Operationssaal [23].

## Diskussion

Auch wenn sich die Weiterbildungen in Urologie im DACH-Raum sehr ähnlich sind, gibt es dennoch auch deutliche Unterschiede.

Zunächst fällt die kürzere Weiterbildungszeit in Deutschland auf (5 vs. 6 Jahre in der Schweiz und in Österreich). Auch entfällt in Deutschland die chirurgische Grundausbildung als obligater Bestandteil. Das ermöglicht es den Weiterzubildenden in Deutschland die gesamte Weiterbildungszeit an einem Standort zu absolvieren. Dies mag für viele ein Vorteil sein, weil z. B. Pendelzeit oder gar ein Umzug wegfallen. Gleichzeitig bietet ein Abteilungswechsel, wie er in Österreich nach der Grundausbildung in Chirurgie und in der Schweiz nach der Basisausbildung und innerhalb der fachspezifischen Ausbildung vorgesehen ist, auch neue Perspektiven und Eindrücke.

Die Deutsche Gesellschaft für Urologie (DGU) hat zur Verbesserung der Weiterbildungsqualität das Weiterbildungscurriculum Urologie (WECU) auf den Weg gebracht. Um an diesem zertifizierten Programm erfolgreich teilzunehmen, ist u. a. eine Beschäftigung für mindestens ein Jahr in einer urologischen Praxis vorgesehen. Dieser Wechsel der Ausbildungsstätte soll ganz bewusst auch zur Horizonterweiterung beitragen [10, 30].

Die Möglichkeit zur Teilzeitarbeit ist in allen Ländern gegeben. Insgesamt unterscheiden sich die Länder aber deutlich in der Gestaltung der Arbeitszeitregelungen. In der Schweiz ist die 45- bis 50-h-Woche oft normal, in Österreich dagegen ist ein Wochenpensum von 35 h vorgegeben. Deutsche Kliniken liegen aufgrund anderer gesetzlicher Regelungen meist dazwischen. Damit werden die Kandidat*innen in der Schweiz deutlich länger am Arbeitsplatz ausgebildet als ihre deutschen und österreichischen Kolleg:innen [9].

Forschung während der Weiterbildung hat für alle Fachgesellschaften des DACH-Raums einen hohen Stellenwert. Dies unterstreicht auch die mögliche Anerkennung von Forschungszeiten während der Weiterbildung. Eine wirkliche „Pflicht“ (Pflichtpublikation, verpflichtende Teilnahmen am Jahreskongress der Fachgesellschaft und dortige Pflichtpräsentationen) zum wissenschaftlichen Arbeiten besteht allerdings nur in der Schweiz. Deutschland und Österreich versuchen auf anderen Wegen, z. B. durch das wissenschaftliche Modul in Österreich oder die zertifizierte strukturierte Weiterbildung nach Vorbild der DGU (WECU) mit möglicher Forschungsfreistellung in Deutschland, der Forschung mehr Raum zu geben [17, 22].

Die Inhalte der Weiterbildungen sind grundsätzlich sehr ähnlich und zielen auf eine breite und allgemeine urologische Ausbildung ab. Österreich bietet mit der Schwerpunktausbildung als einziges Land bereits in der Weiterbildung eine Spezialisierung an. Deutschland und Österreich machen weniger Vorgaben zur Vertiefung bestimmter Gebiete.

Logbücher sind in allen Ländern obligat. Auch Richtzahlen finden sich in allen Weiterbildungsordnungen. Evidenz dafür, worauf diese Zahlen beruhen, bleiben jedoch alle zuständigen Organe schuldig. Weiter wird wohl nur selten nachgeprüft, ob die bestätigten Zahlen wirklich der Realität entsprechen [16]. Die neue Weiterbildungsordnung in Deutschland geht einen Schritt weiter und weg vom reinen „Absitzen“ einer bestimmten Zeit und „Abhaken“ von Themen und Zahlen, hin zur kompetenzbasierten Weiterbildung [5, 8].

Mit der Einführung von Kompetenzniveaus wurde ein Schritt weg von starren und willkürlich wirkenden Zahlenwerken hin zu einer realistischeren Beurteilung der Weiterzubildenden gemacht. Zwar müssen die Kompetenzniveaus und Zahlenvorgaben weiterhin von beiden Seiten „abgehakt“ werden, dennoch haben die neuen Kompetenzniveaus deutlich höheres Potenzial zu konstruktivem Feedback, da sie Wissen und Fähigkeiten genauer definieren als reine Zahlen [7].

Was die Gestaltung der Prüfungen angeht, gibt es in den DACH-Ländern noch deutlichen Verbesserungsbedarf. Deutschland und Österreich begnügen sich mit jeweils einer summativen Prüfung zur Erlangung des Facharzttitels. Die Schweiz ist hier etwas progressiver und schreibt zumindest vier verpflichtende formative Prüfungen pro Jahr vor. Ob diese dann wirklich durchgeführt und als Feedback-Instrument genutzt werden, hängt jedoch von der lokalen Ausbildungskultur ab [11]. Mit den zwei zusätzlichen verpflichtenden summativen Prüfungen liegen die Anforderungen an die Weiterzubildenden in der Schweiz im Dreiländervergleich mit Abstand am höchsten.

Eine regelhafte Qualitätskontrolle der Weiterbildung erfolgt in Deutschland und Österreich nicht, die Schweiz führt regelmäßige Evaluationen durch [3].

Da die Facharztbezeichnung in allen DACH-Ländern unbefristet verliehen wird und (bis auf CME-Punkte) keine wiederkehrenden Qualifikationsnachweise vonnöten sind, ist die Frage durchaus berechtigt, ob die Prüfungsart, -anzahl und -form gerade in Deutschland und Österreich noch zeitgemäß sind. Zum Vergleich: Kolleg:innen in den Vereinigten Staaten müssen sich spätestens alle 10 Jahre erneut zertifizieren lassen.

Schaut man über den Tellerrand und die DACH-Grenzen hinaus, entwickeln sich zahlreiche Weiterbildungscurricula hin zu weniger zeit- und zahlenbasierten Modellen. Mit der Einführung von EPA und deren Nutzung für die Curriculumsentwicklung ergab sich ein rasanter Wandel in zahlreichen Bereichen der medizinischen Ausbildung weltweit [2]. Im deutschen Sprachgebrauch werden EPA häufig auch als anvertraubare professionelle Tätigkeiten (APT) bezeichnet [1]. EPA beschreiben Arbeitspakete oder Tätigkeiten, welche im Arbeitsalltag notwendig sind, Kompetenzen hingegen macht sich der/die Weiterzubildende zu eigen. EPA aus der Urologie könnten z. B. die Betreuung einer/s Ambulanzpatient*in, bestimmte Operationen oder die Kommunikation mit Kolleg*innen, Patient*innen und Angehörigen sein. Definierte EPA bieten damit die Möglichkeit zur Erstellung individualisierter Weiterbildungspläne und haben zudem das Potenzial, Feedback zu fördern [15]. In der Schweiz hat der Wandel mit der Einführung von EPA im Rahmen des PROFILES-Projekts während des Humanmedizinstudiums bereits begonnen [13]. Im Bereich der postgraduierten Weiterbildung ist das Konzept hingegen noch nicht vollständig angekommen. Hier könnte die Urologie eine Vorreiterrolle einnehmen.

## Fazit für die Praxis

- Ein Länderwechsel im DACH-Raum (D = Deutschland, A = Österreich, CH = Schweiz) ist während der Weiterbildung ohne große Hürden möglich.
- Die Weiterbildung dauert in Deutschland mindestens 5, in Österreich und der Schweiz 6 Jahre.
- In Österreich und der Schweiz ist die chirurgische Grundausbildung obligat.
- Eine Spezialisierung während der Weiterbildung ist nur in Österreich fest vorgesehen.
- Die Anerkennung von Forschungszeit ist in allen Ländern möglich.
- Nur in der Schweiz sind formative Prüfungen verpflichtend. In allen Ländern ist mindestens eine summative Prüfung (schriftlich und/oder mündlich) notwendig.
- In der Schweiz ist der Zeitaufwand für die Weiterbildung am größten, die Weiterbildung muss an verschiedenen Einrichtungen erfolgen, insgesamt werden die modernsten Methoden der Qualitätskontrolle genutzt. Zudem muss mindestens eine wissenschaftliche Publikation vorgelegt werden.
- Die Weiterbildungsordnung von 2020 in Deutschland nimmt Kompetenzniveaus auf. Zudem bietet die DGU mit dem Weiterbildungscurriculum neuerdings eine deutlich verbesserte Struktur an.
- International gewinnen EPA-basierte („entrustable professional activities“) Curricula an Bedeutung, die DACH-Länder hinken hier hinterher, die Urologie könnte eine Vorreiterrolle einnehmen.
